# De novo collapsing glomerulopathy after kidney transplantation: Description of two cases 

**DOI:** 10.5414/CNCS110887

**Published:** 2023-04-19

**Authors:** Roberta Cutruzzulà, Selene Laudicina, Alfredo Bagalà, Leonardo Caroti, Marilù Bartiromo, Iacopo Gianassi, Luciano Moscarelli, Lorenzo Di Maria, Aida Larti, Marco Allinovi, Giulia Antognoli, Calogero L. Cirami

**Affiliations:** 1 Nephrology Unit, Careggi University Hospital, and; 2 Department of Experimental and Clinical Medicine, University of Florence, Florence, Italy

**Keywords:** collapsing glomerulopathy, focal segmental glomerulosclerosis, kidney transplantation, proteinuria, graft

## Abstract

Background: Among different forms of de novo focal segmental glomerulosclerosis (FSGS), which can develop after kidney transplantation (KTx), collapsing glomerulopathy (CG) is the least frequent variant, but it is associated with the most severe form of nephrotic syndrome, histological findings of important vascular damage, and a 50% risk of graft loss. Here, we report two cases of de novo post-transplant CG. Clinical presentation: A 64-year-old White man developed proteinuria and worsening of renal function 5 years after KTx. Before the KTx, the patient was affected by an uncontrolled resistant hypertension, despite multiple antihypertensive therapies. Blood levels of calcineurin inhibitors (CNIs) were stable, with intermittent peaks. Kidney biopsy showed the presence of CG. After introduction of angiotensin receptor blockers (ARBs), urinary protein excretion progressively decreased in 6 months, but subsequent follow-up confirmed a progressive renal function decline. A 61-year-old White man developed CG 22 years after KTx. In his medical history, he was hospitalized twice to manage uncontrolled hypertensive crises. In the past, basal serum cyclosporin A levels were often detected above the therapeutic range. Low doses of intravenous methylprednisolone were administered due to the histological inflammatory signs shown on renal biopsy, followed by a rituximab infusion as a rescue therapy, but no clinical improvement was seen. Discussion and conclusion: These two cases of de novo post-transplant CG were supposed to be mainly caused by the synergic effect of metabolic factors and CNI nephrotoxicity. Identifying the etiological factors potentially responsible for de novo CG development is essential for an early therapeutic intervention and the hope of better graft and overall survival.

## Introduction 

Collapsing glomerulopathy (CG) is one of the variants of focal segmental glomerulosclerosis (FSGS), as stated in the KDIGO classification. FSGS can affect both native and transplanted kidneys. In the latter case, FSGS can represent a recurrent or de novo disease. Current KDIGO guidelines suggest a stratification of FSGS in primary, genetic, secondary, and undetermined cause (FSGS-UC) [1]. Primary FSGS has been attributed to a circulating permeability factor that has not yet been identified. Currently, the only form of FSGS that can be reasonably attributed to a circulating permeability factor is the FSGS that recurs rapidly after a kidney transplant, and which can be successfully treated by plasmapheresis [2, 3, 4, 5]. FSGS-UC occurs in the absence of a genetic or identifiable secondary cause and in the absence of clinical and histologic manifestations of primary FSGS. Genetic FSGS does not typically recur after kidney transplantation (KTx). Secondary FSGS lesion is found in the setting of an established pathophysiologic process known to cause FSGS. De novo FSGS is usually diagnosed 1 year after transplantation [6], and etiological factors range from calcineurin inhibitor (CNI) toxicity, viral infections, hypertension, and immunological injury [7, 8]. Among different forms of de novo post-transplant FSGS, CG is the least frequent variant, but it is associated with a more severe nephrotic syndrome, histological findings of increased vascular damage, and a significant risk of graft loss (up to 50% of cases) [9]. Here, we report two cases of de novo CG that developed after KTx, which are believed to be mainly caused by the synergic effect of metabolic factors and CNI nephrotoxicity. 

## Case report 

### Patient A 

**Figure 1.**
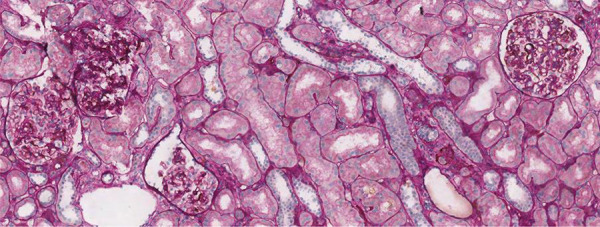
Light microscopy of patient A. Two glomeruli show segmentary sclerosis of the flocculus. Focal aspect of collapse of glomerular capillaries is shown in a glomerulus with some hyperplastic epithelial cells containing drops of protein reabsorption (collapsing lesion). No signs of endocapillary hypercellularity or glomerulitis.

**Figure 2.**
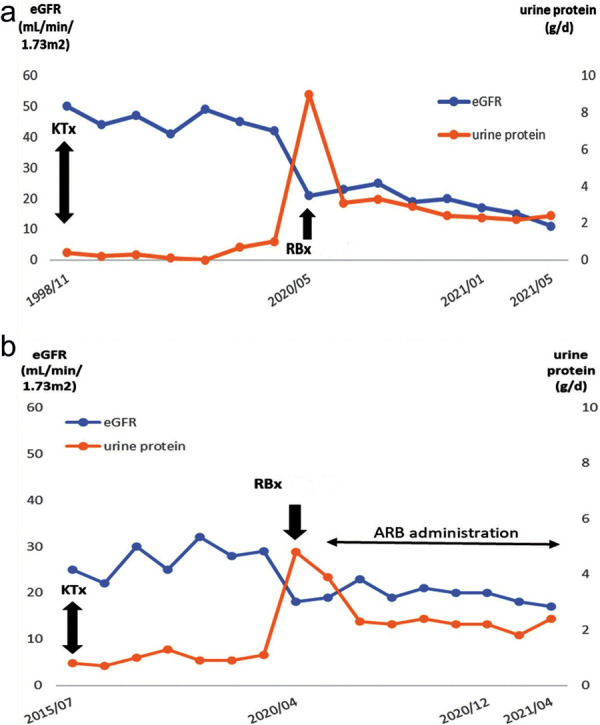
Clinical course after transplantation. eGFR = estimated glomerular filtration rate; KTx = kidney transplantation; RBx = renal biopsy.

A 64-year-old White man was affected by end-stage kidney disease from the age of 30, probably secondary to hypertensive nephrosclerosis, although a kidney biopsy was not performed on native kidney. No pediatric history of infections, vesicoureteral reflux, or known glomerulonephritis was reported, and no smoking history either. Genetic analysis was not performed at that time. He presented a history of uncontrolled severe arterial hypertension despite different antihypertensive drugs (angiotensin receptor blocker (ARB), calcium channel blocker, β-blocker, α-adrenergic and imidazoline receptor agonist) complicated by hypertension-related cardiac and ocular diseases, insulin-dependent type 2 diabetes mellitus, obesity (BMI 30 kg/m^2^), and peripheral arterial disease. He underwent KTx from a cadaveric donor at the age of 59. Serological tests by complement-dependent cytotoxic (CDC) crossmatch were negative, and human leukocyte antigen (HLA) typing revealed 2 mismatches in their A, B, DR loci. Immunosuppressive therapy included cyclosporin A (CsA), mycophenolate mofetil (MMF), prednisolone, and induction therapy with anti-interleukin-2 receptor (anti-IL 2R). After 3 years, CsA and MMF were switched to tacrolimus (FK-506) and azathioprine (AZA) due to gastrointestinal disorders. At 3 months after KTx, a proximal ureteral stenosis occurred requiring stenting. No other perioperative complications were observed. Baseline serum creatinine (sCr) level after KTx was 2.7 mg/dL (eGFR 25 mL/min/1.73m^2^), and urinary protein excretion was 0.8 g/d. Abdominal ultrasound showed a regular-sized graft, with no signs of obstruction or macrovascular damage. During follow-up, initial concentrations of CsA and subsequent concentrations of tacrolimus were generally within the therapeutic range, with some intermittent peaks, while anti-donor specific antibody (DSA) typing was negative. Home blood pressure measurements identified an average 160/90 mmHg despite multiple antihypertensive drugs, and control of metabolic syndrome did not improve. Five years after KTx, sCr and urinary protein excretion gradually increased (sCr 3 mg/dL, urinary protein 4.8 g/d). We performed a transplant renal biopsy that revealed no signs of rejec- tion, no segmental floccular sclerosis and no focal aspects of collapse of glomerular capillaries. Moderate arteriolar hyalinosis and intimal fibrosis were also described, possibly reflecting CNI-associated arteriopathy. Ultrastructural examination revealed extensive podocyte foot processes effacement (< 90%) in the absence of immune deposits (Figure 1). Diagnosis of collapsing glomerulopathy was confirmed. Screening for secondary causes of collapsing FSGS (HIV, HBV, HCV, parvovirus, SARS-CoV-2, urinary cytology, chest radiography, and colonoscopy) permitted the exclusion of infections and tumors. After administration of ARBs, urinary protein excretion decreased to 2.2 g/d in 6 months, but subsequent follow-up confirmed a progressive renal function decline (sCr 3.6 mg/dL, eGFR 17 mL/min/1.72m^2^) 12 months after the biopsy (Figure 2a). Immunosuppressive therapy was not changed after CG diagnosis. Moreover, uncontrolled blood pressure and overweight persisted, supporting a de novo CG mainly caused by vascular/metabolic factors and CNI nephrotoxicity. 

### Patient B 

A 61-year-old White man received a first diagnosis of advanced chronic kidney disease (stage 4 CKD, baseline eGFR 19 mL/min/1.72m^2^ and sCr 4 mg/dL) at the age of 34. At disease onset, the patient suddenly experienced fever, gastrointestinal symptoms (nausea, vomiting, diarrhea, abdominal pain), myalgias, and high blood pressure level (200/100 mmHg). There was no family history of kidney disease, diabetes mellitus, or obesity. Suspected abuse of non-steroidal anti-inflammatory drugs (NSAIDs) was reported. Genetic analysis and renal biopsy were not performed at that time, so the initial native kidney disease was unknown. After 3 years of hemodialysis therapy, at the age of 39, he received a KTx from a cadaveric donor. The HLA typing revealed 2 mismatches in their A, B, DR loci. Immunosuppressive therapy included CsA, AZA, and prednisolone. AZA was stopped after 3 months because of recurrent urinary tract infections. Baseline sCr level was 1.7 mg/dL (eGFR 50 mL/min/1.73m^2^), and urinary protein excretion was 0.4 g/d. Medical history was also characterized by an uncontrolled severe arterial hypertension on multiple antihypertensive therapies (angiotensin-converting enzyme inhibitors, β-blocker, α-adrenergic and imidazoline receptor agonists), complicated by heart disease and ocular retinopathy. Moreover, basal serum CsA levels were often above the therapeutic range. Anti-HLA DSA detection was negative. In May 2020, over 20 years after receiving KTx, the patient suddenly contacted our transplant center complaining of general discomfort and dyspnea. On clinical examination, he presented weight gain, edema in the lower limbs, uncontrolled blood pressure, and an important hypervolemic status. sCr and blood urea levels were significantly increased (sCr 3 mg/dL, urea 170 mg/dL), and urinary protein excretion was 9 g/d. Basal serum CsA levels were 150 ng/mL, serological tests detecting PCR DNA of CMV, EBV, JC, and BK were negative, together with anti-HLA DSA antibodies. SARS-CoV-2 infection was ruled out by molecular swab. Abdominal ultrasound showed a reduced kidney graft size with reduced cortico-medullary differentiation, with no signs of obstruction or macrovascular damage. The renal biopsy showed glomerular tuft retraction and epithelial cell proliferation in 3 – 4 glomeruli, focal areas of mesangial proliferation, and thickening of the glomerular basement membrane. Moderate fibrous thickening in the medium vessels and intimal hyalinosis in the small vessels were described. The diagnoses of CG and CsA associated arteriolopathy (CAA) were confirmed. 

Screening for secondary causes of collapsing FSGS (HIV, HBV, HCV, SARS-CoV-2, parvovirus, urinary cytology, chest radiography, colonoscopy) permitted the exclusion of infections and tumors. Genetic testing was also performed and showed no suspicious variants. To switch off histological inflammatory signs shown on renal biopsy, low doses of intravenous methylprednisolone were administered. Moreover, despite the lack of evidence in the literature of efficacy in this setting, but considering the rapid onset and progression of the disease, we administered an infusion of rituximab (1 g) as a rescue therapy, supported by the favorable role of rituximab on podocyte apoptosis and cytoskeleton integrity [10, 11, 12, 13]. As expected, in absence of further clinical improvement, we decided to administer a conservative therapy by lowering the dose of cyclosporine of 10 mg every 2 weeks. After 12 months from the biopsy, the patient is still on conservative medical therapy. Renal function is settled on stage 5 CKD (sCr 4.2 mg/dL, eGFR 11.5 mL/min/1.73m^2^). Blood pressure is still poorly controlled despite multiple antihypertensive drugs, the body weight has progressively increased (BMI 30 kg/m^2^), but proteinuria levels have dropped to 2.2 g/d (Figure 2b). 

## Discussion 

The reason for the late onset of de novo FSGS after KTx remains to be clarified. Incidence of de novo FSGS is 1 – 9% in renal allografts [14, 15, 16], and the pathogenesis is largely unknown. Although renal disease progression is slower in de novo FSGS than in the recurrent disease, the prognosis after KTx is poor, and CG is associated with a worse prognosis [13, 17, 18]. CG has been reported in several clinical conditions: longstanding grafts (in which nephron loss and fibrosis have resulted in glomerular hyperfiltration injury), donor-recipient size-mismatched kidney grafts (in whom the presumed pathogenesis is hyperfiltration injury), grafts with severe vascular disease (resulting in presumed glomerular hypofiltration leading to secondary CG), grafts with chronic transplant glomerulopathy (in which immunological chronic glomerular endothelial and podocyte injuries may develop with segmental FSGS aspects), and grafts with chronic CNI-induced glomerulopathy (in which chronic glomerular endothelial and podocyte injury may be induced by CNI-associated arteriopathy). In general, arterial hypertension, immunosuppressive therapies, diabetes mellitus, immunological dysfunction, and infections become important contributing factors in the pathogenesis of post-transplant FSGS [5]. Regarding CNI-associated FSGS, cumulative evidence has suggested that FSGS occurrence rate increased after CNI introduction from 1.5% in the prior immunosuppressant era to 10.5% in the CsA era. Moreover, late-onset de novo FSGS after KTx has shown strong association with severe CAA [7, 19, 20, 21]. We also found this association in our 2 patients, who showed concomitant CAA on histological examination. By increasing vasoconstrictor factors and reducing vasodilatory factors, CNIs can lead to podocyte injury with consequent detachment from the glomerular basement membrane and glomerulosclerosis. Additionally, CNIs can increase the expression of TGF-β in podocytes, leading to podocyte apoptosis, synechia, and sclerosis [22, 23, 24]. In case B, we chose a more aggressive therapy supported by recent research that showed that rituximab can act directly on podocytes, stabilizing the cytoskeleton and improving proteinuria through a B-cell-independent mechanism [10, 11, 12, 13]. Hypertension, diabetes, immune-mediated injury, BK polyoma virus, or parvovirus B19 infections [5, 25] and any other condition leading to loss of renal mass can be involved in the pathogenesis of FSGS. In a study by Patel et al. [5], hypertension was the strongest associated finding in their patients with de novo FSGS, with 100% of patients on anti-hypertensive agents. In our two cases, uncontrolled hypertension can also be considered the strongest associated factor to the de novo CG. Both patients were on multiple antihypertensive therapies, often including four different drugs. Various studies have shown an association between immunological abnormalities or rejection and post-transplantation FSGS [6, 8, 9, 10, 11, 12, 13, 14, 26]. In our patients, negative findings of anti-DSA and C4d staining in allograft biopsies could rule out a role of immunological aspects to the development of FSGS. However, these observations are not sufficient to reasonably rule out subclinical immune-mediated damage to the graft, such as subclinical rejection (SCR), often demonstrable by protocol biopsies. Moreover, protocol biopsies are a valid support tool for early detection of CNI toxicity. In our cases, we cannot exclude subclinical immunological changes as hypothetical potential risk factors for the de novo CG. In conclusion, we observed an improvement of proteinuria and a stabilization of kidney function in both cases. Despite the decision to treat patient B with a low dose of steroids and rituximab, retrospective studies or case series suggest that only conservative therapy can delay the worsening of kidney function in this subset of patients. Further studies would be needed to show the impact of protocol biopsies for early detection of potential CNI toxicity and consequent early switching to other immunosuppressive therapies [27]. Nevertheless, it is difficult for such studies to come to light, given the rarity of the FSGS after KTx. 

## Conclusion 

Among de novo post-transplant FSGS forms, CG is the least frequent variant, and its prognosis is still poor. CG is associated with more severe vascular changes, higher degree of proteinuria, more frequent renal insufficiency, and a higher rate of graft loss [9]. It is strongly important to identify the underlying etiological factors responsible for post-transplant CG development in order to correct them early by starting therapeutic interventions for a consequent better graft and patient survival. In post-transplant follow-up, strict control of CNIs blood levels, arterial pressure, BMI, glucose and lipid metabolism are necessary to prevent graft damage related to these factors. Protocol biopsies may represent an important strategy for the early detection of CNI-associated nephrotoxicity and subclinical rejection in order to optimize/switch immunosuppressive therapies and improve the short- and long-term survival of the graft. 

## Funding 

This work has not received any contribution, grant or scholarship. 

## Conflict of interest 

The authors have no conflict of interest to declare.
